# The Role of Pulmonary and Systemic Immunosenescence in Acute Lung Injury

**DOI:** 10.14336/AD.2017.0902

**Published:** 2018-08-01

**Authors:** Christina Brandenberger, Katharina Maria Kling, Marius Vital, Christian Mühlfeld

**Affiliations:** ^1^Institute of Functional and Applied Anatomy, Hannover Medical School, Hannover, Germany; ^2^Biomedical Research in Endstage and Obstructive Lung Disease Hannover (BREATH), Member of the German Center for Lung Research (DZL), Hannover, Germany; ^3^Cluster of Excellence REBIRTH (From Regenerative Biology to Reconstructive Therapy), Hannover, Germany; ^4^Microbial Interactions and Processes Research Group, Helmholtz Centre for Infection Research, Braunschweig, Germany

**Keywords:** acute lung injury, immunosenescence, alveolar macrophages, pulmonary inflammation, systemic inflammation, lipopolysaccharide

## Abstract

Acute lung injury (ALI) is associated with increased morbidity and mortality in the elderly (> 65 years), but the knowledge about origin and effects of immunosenescence in ALI is limited. Here, we investigated the immune response at pulmonary, systemic and cellular level in young (2-3 months) and old (18-19 months) C57BL/6J mice to localize and characterize effects of immunosenescence in ALI. ALI was induced by intranasal lipopolysaccharide (LPS) application and the animals were sacrificed 24 or 72 h later. Pulmonary inflammation was investigated by analyzing histopathology, bronchoalveolar lavage fluid (BALF) cytometry and cytokine expression. Systemic serum cytokine expression, spleen lymphocyte populations and the gut microbiome were analyzed, as well as activation of alveolar and bone marrow derived macrophages (BMDM) *in vitro*. Pulmonary pathology of ALI was more severe in old compared with young mice. Old mice showed significantly more inflammatory cells and pro-inflammatory cyto- or chemokines (TNFα, IL-6, MCP-1, CXCL1, MIP-1α) in the BALF, but a delayed expression of cytokines associated with activation of adaptive immunity and microbial elimination (IL-12 and IFNγ). Alveolar macrophages, but not BMDM, of old mice showed greater activation after *in vivo* and *in vitro* stimulation with LPS. No systemic enhanced pro-inflammatory cytokine response was detected in old animals after LPS exposure, but a delayed expression of IL-12 and IFNγ. Furthermore, old mice had less CD8^+^ T-cells and NK cells and more regulatory T-cells in the spleen compared with young mice and a distinct gut microbiome structure. The results of our study show an increased alveolar macrophage activation and pro-inflammatory signaling in the lungs, but not systemically, suggesting a key role of senescent alveolar macrophages in ALI. A decrease in stimulators of adaptive immunity with advancing age might further promote the susceptibility to a worse prognosis in ALI in elderly.

The performance of the immune system declines with advancing age and evokes a greater susceptibility to infections and inflammatory diseases in the elderly (< 65 years). This phenomenon is known as immune-senescence and includes age-related disparities in innate and adaptive immunity [1] such as decreased phagocytotic function of macrophages and neutrophils, enhanced serum levels of pro-inflammatory cytokines, more airway neutrophils or decreased T-cell stimulation by dendritic cells [2].

Severe pulmonary inflammation or sepsis can result in acute lung injury (ALI) or *Acute Respiratory Distress Syndrome* (ARDS). ALI is characterized by inflammatory cell infiltrates and damage of the air-blood barrier, leading to pulmonary edema, hypoxemia and potentially respiratory failure [3]. Multiple factors are involved in the development of the disease and although the first description of ALI was 50 years ago, there is still no effective pharmaceutical treatment available and the main therapeutic strategy to prevent respiratory failure remains mechanical ventilation [4,5]. Particularly elderly patients with ARDS have a decreased survival rate [6-8], as the mortality of patients older than 55 years of age is significantly higher than in younger patients [8] and survivors with an age of > 70 years have more difficulties to recover [7]. It is supposed that comorbidities and immunosenescence are mainly responsible for the increased morbidity and mortality rate in the elderly, but underlying mechanisms are still poorly understood.

In a previous study we investigated alterations in pulmonary structure, function and inflammation in young and old mice in a lipopolysaccharide (LPS) model of ALI within 24 h [9]. ALI was more severe in old compared with young mice, resulting in enhanced pulmonary and septal edema, altered lung function and greater pulmonary neutrophil recruitment that contribute to damage in the air-blood barrier. Other age-related investigations in murine models of ALI, induced with bacteria or bacterial components, also provided evidence of a greater pulmonary inflammation in old animals, with enhanced neutrophil numbers and inflammatory cytokine levels [10-13]. Furthermore old septic mice were shown to have a reduced survival rate compared with young animals [14]. Hence the findings in murine models of ALI concede with those in elderly patients with ARDS.

While a greater inflammatory response and severity in ALI has been recognized with progressing age, it is not clear what drives the enhanced inflammation. Potential contributors of enhanced inflammation in ALI of the elderly could be a chronic systemic inflammation [15], changes in pulmonary function and physiology [16], a decline in anti-inflammatory response [13] or changes in abundance or performance of particular immune cells [17]. For optimal treatment approaches in elderly patients it is, however, particularly important to understand what kind of age-related changes in immune response contribute to the severity of the disease. In the current study we therefore investigated the pulmonary and systemic inflammatory response in young (2-3 months) and old (18-19 months) C57BL/6J mice to further localize the potential source of enhanced inflammatory reaction in old animals. As we anticipated temporal changes in development or resolution of inflammation in old compared with young mice, the animals were sacrificed 24 h or 72 h after induction of ALI. In LPS-models of ALI the pro-inflammatory response evolves within the first 24 h and at least some inflammatory parameters are expected to decline or resolve again after 72 h [18].

The pulmonary inflammation was assessed by analyzing histopathology, bronchoalveolar lavage fluid (BALF) cytometry and cytokine expression. The systemic inflammation was investigated by analyzing serum cytokines, spleen lymphocyte populations including regulatory T-cells (T-regs), natural killer cells (NK cells) and CD8^+^ T-cells. The gut microbiome, as known to shape lymphocyte populations and the immune system beyond the gut [19,20], was analyzed as well. We further hypothesized that macrophages are a main source of the enhanced pro-inflammatory response in ALI of old mice, as they are some of the first cells to activate the immune system. For this reason an age-depended activation and cytokine production of alveolar macrophages (AM) was analyzed after *in vitro* and *in vivo* stimulation as well as *in vitro* stimulation of bone marrow derived macrophages (BMDM) that resemble more the systemically recruited macrophages in their phenotype.

## MATERIAL AND METHODS

### Animal model and necropsy

Young and old male C57BL/6Jrj mice were purchased from Janvier Labs (France) at an age of 10 weeks and 18 months, respectively. The animals were allowed to acclimate in the local housing facility (Hannover Medical School animal facility) for at least one week before experimental start. All animal procedures were approved by the Lower Saxony State Office for Consumer Protection and Food Safety (LAVES) in accordance with German law for animal protection and with the European Directive, 2010/63/EU. ALI was induced by intranasal (i.n.) administration of 2.5 mg/kg body weight LPS (LPS-mice; n=10 per age and exposure group) or saline (control mice; n=7 age and exposure group) as described previously [9]. The animals were sacrificed 24 and 72 h post-treatment. Blood serum and bronchoalveolar lavage fluid (BALF) was collected. The lungs were excised, processed for histopathology and the spleens digested and stained for FACS analysis. Caecal content was collected at necropsy, snap frozen in liquid nitrogen and stored at -80°C. During the experiments, two of the old LPS-treated animals died between day 2 and 3 post-treatment. Two additional old animals were therefore included into the study. Although all animals seemed healthy at the start of the experiments, it cannot be excluded that some mice, particularly the old ones, may have suffered from pre-existing comorbidities.

### Histopathology

The left lung lobe of each animal was chemically fixated by intra-tracheal instillation with 4% paraformaldehyde at a constant pressure of 25 cm H_2_O for 1 h and then stored for 4-7 days in fixative. The lobe was randomly cut in sections and embedded in paraffin. Histological sections were stained with hematoxylin and eosin (H&E).

### BALF Cytometry and BALF Protein

BALF and serum were collected as described previously [9]. Differential cell counts were calculated, and total BALF-protein was measured by using the Pierce BCA Protein Assay Kit (Thermo Scientific, USA) according to the provider’s manual. BALF protein and cell differentials (total cells, neutrophils and macrophages) from mice with 24 h LPS exposure were presented previously in Kling et al. [9]. BALF macrophages were further subject to characterization by flow cytometry. BALF macrophage samples of two animals from the same exposure group were pooled in the 24 h exposure experiment (resulting in n = 3-5). However, at the 72 h time point all BALF samples were tested individually. BALF cells were first blocked with anti-CD16/32 (BioLegend) and 5% fetal calf serum (FCS) and then stained for 30 min on ice with antibodies from BioLegend (CD11b-PerCP/Cy5.5, CD11c-FITC, CD11c-APC, CD80-PE, CD86-APC, CD86-FITC) and BD Biosciences (SiglecF-APC/Cy7) and measured with a FACS Canto II flow cytometer (BD Biosciences). Alveolar macrophages were selected as CD11c^+^, SiglecF^+^ and CD11b^low^ cells and expression of CD80 and CD86 was assessed as mean fluorescent intensity (MFI).

### In vitro stimulation of alveolar and bone marrow derived macrophages

AM were harvested from the BALF of untreated young and old mice (n = 4 per experimental group). The cells were washed, counted, diluted to a concentration of 2.5 x 10^5^ cells/mL and seeded in a 96-well culture dish. The AMs were allowed to adhere to the culture wells for 4 h in the incubator, then non-adhered cells were washed off and AMs were cultured in DMEM medium (Gibco) plus 1% Pen/Strep and 10% FCS (controls) or medium plus supplement with 100 ng/mL LPS for 24 h. After stimulation, cells and supernatant were harvested. The AMs were stained and analyzed with the same protocol as for the *in vivo* studies.

BMDMs were isolated from young and old mice and differentiated for 7 days in culture with 10 ng/mL M-CSF as described previously [21]. A number of n = 6 bone marrow isolations per age group were performed. Cells were then cultured in 12-well plates in F12/DMEM medium (Gibco) plus 1% Pen/Strep and 10% FCS and stimulated *in vitro* with 100 ng/mL LPS for 24 h. Afterwards the cells were harvested and stained for flow cytometric measurements with CD11b-PerCP/Cy5.5, F4/80-APC, CD80-PE, CD86-FITC (BioLegend).

### Cytokine analysis

Cytokine levels of the BALF and cell culture supernatant were assessed with the 13 plex mouse inflammation kit and the chemokine mouse kit for KC and MIP-1α (BioLegend, Germany; sensitivity 5 pg to 10 ng/mL). Cytokines in the blood serum were analyzed with the enhanced sensitivity FLEX SET (200 fg to 200 pg/mL) Cytometric Bead Array (BD Biosciences, Germany). Both analyses were performed according to the supplier’s manual and measured with a FACS Canto II flow cytometer (BD Biosciences).

### Spleen cell analysis

Spleens were excised at necropsy and processed as previously described [22]. Total splenocyte populations were counted and stained with antibodies from BioLegend (CD3-FITC, CD8-APC, CD4-APC/Cy7, CD44-APC, CD62L-PE, CD25-PE/Cy7, NK1.1-PE/Cy7, CD11b-PerCP/Cy5.5, CD27-PE) for 30 min on ice. Measurements were performed with a Canto II flow cytometer (BD Biosciences). CD8^+^ T-cells were selected as CD3^+^, CD8^+^ and CD4^-^, regulatory T-cells (T-regs) were selected as CD3^+^, CD8^-^, CD4^+^ and CD25^+^ and NK cells were selected as CD3^-^, NK1.1^+^ with CD11b^low/high^ and CD27^low/high^ populations.

### Microbiome analysis

DNA extraction was performed using the PowerSoil kit (MoBio, Jefferson City, MO, USA) according to the manufacturer. Primers for the V1-2 region, amplification and sequencing strategy is described in [23]. Raw reads were merged, filtered for a size >250 nucleotides and subjected to RDP’s classifier for taxonomic classifications (confidence cut-off of 80% [24]). Subsequent analyses were done on the genus level based on rarefied data (3,640 sequences per sample) applying the function *rarefy_even_depth (rngseed=TRUE)* from R’s package *phyloseq* (v. 1.10.0; [25]), except for the *Firmicutes* to *Bacteroidetes* ratio that was calculated on the phylum level. Non-metric multidimensional scaling (NMDS) and permutational ANOVA were performed in R (package: *vegan* (v. 2.3-4) on Hellinger transformed data. FDR corrected bootstrapped Mann-Whitney U tests were performed in Qiime (v. 1.9.1) considering genera detected in ≥25% of samples as suggested by the developers [26].

### Statistical Analysis

Grubbs outlier test was performed and significant outliers (maximum one per experimental group) were excluded from the data set. Differences among groups were analyzed by a two-way analysis of variance (ANOVA) followed by a pair-wise comparison with Bonferroni-test. Data that were not normally distributed were subject to ln or square root transformation prior to data analysis. Significance was assigned to *p* values ≤ 0.05. All analyses were conducted using SigmaPlot software (SYSTAT Software Inc; San Jose, USA).

**Figure 1:**
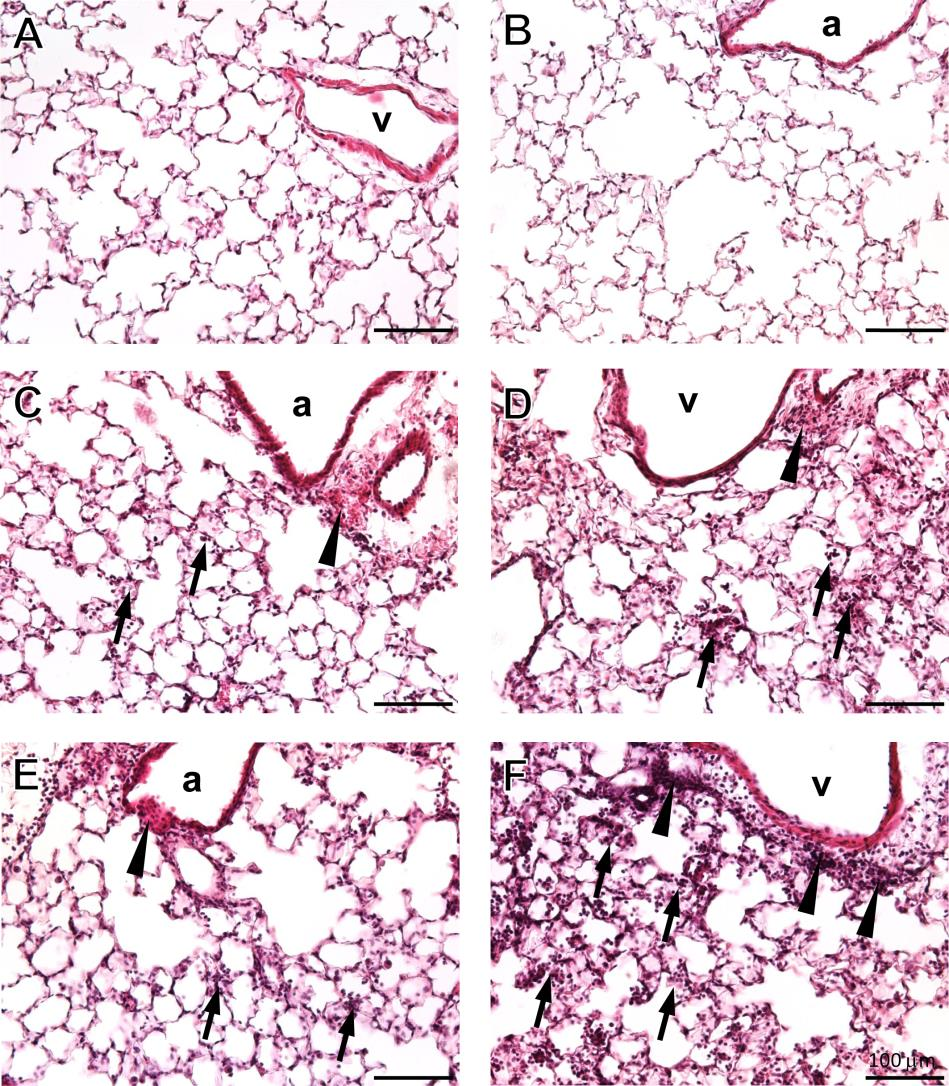
Pulmonary histopathology Representative micrographs of pulmonary histological sections stained with H&E. The lung parenchyma of young (**A**) and old control animals (**B**) do not show any inflammatory cells. Cell infiltrates, such as neutrophils and macrophages were found 24 h and 72 h after LPS-treatment in young (**C, E**) and old LPS (**D, F**) mice, respectively. Arrows indicate inflammatory cell infiltrates in the parenchyma and arrow heads in the peribronchiolar and -vascular area. Inflammatory cells were more pronounced in old compared with young LPS-mice and increased over time. Scale bar = 100 µm. a = airway, v = blood vessel

**Figure 2:**
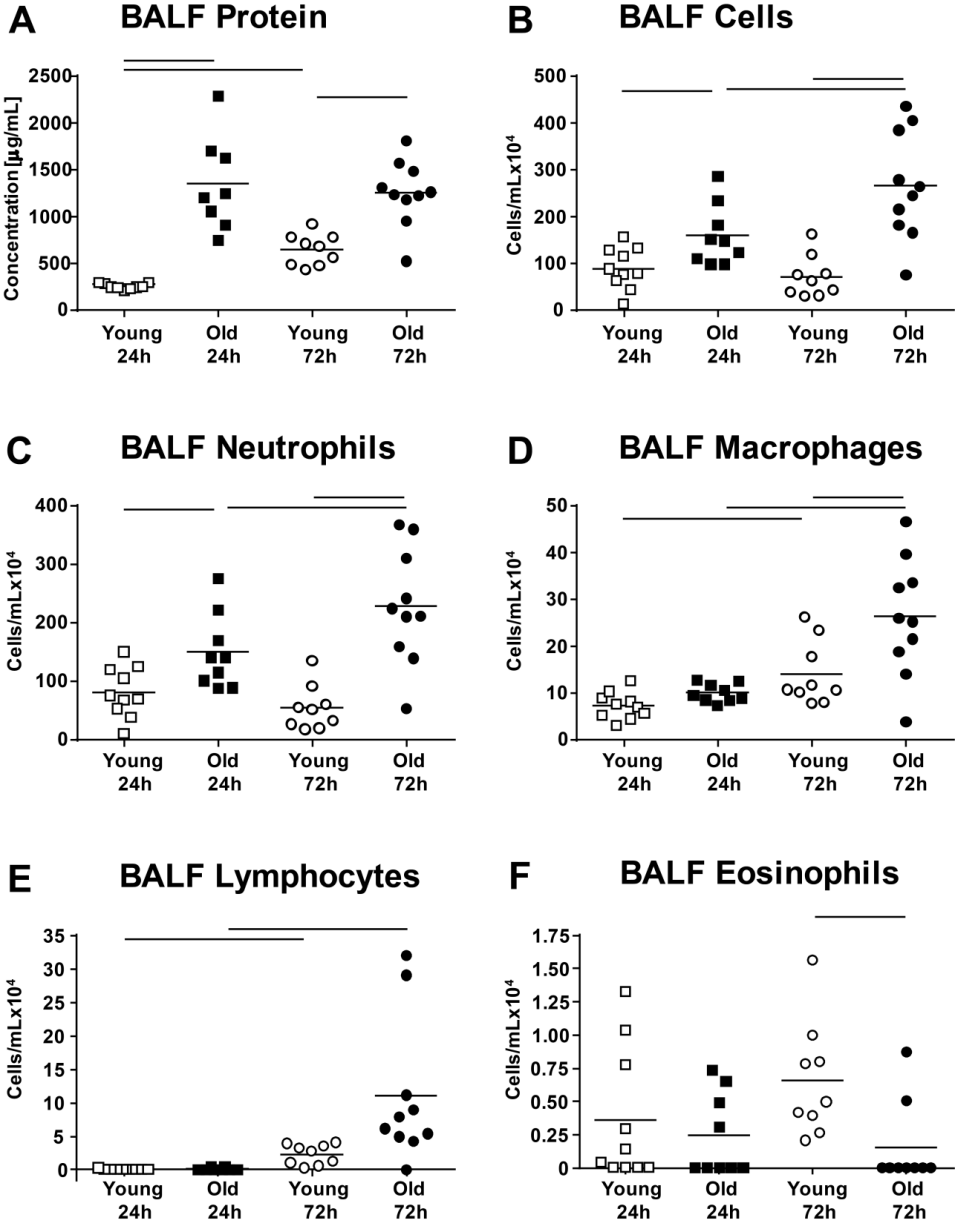
Protein concentration and differential cells counts in the BALF Protein concentration (**A**), total cells (**B**), neutrophils (**C**), macrophages (**D**), lymphocytes (**E**) and eosinophils (**F**) were assessed in the BALF. BALF protein concentration, total BALF cells and BALF neutrophil numbers were greater after 24 h of LPS exposure in old mice (data published in Kling et al. 2017 [9]) and after 72 h of exposure time the numbers of BALF macrophages were also induced in old compared with young mice. Numbers of BALF lymphocytes increased from 24 h to 72 h in young and old mice. Detection of BALF eosinophils was marginal. Each data point represents one animal; means are expressed by horizontal bars; lines indicate statistically significant (*p* < 0.05) differences between groups.

**Table 1:** BALF and serum cytokines.

A) BALF Cytokines	Young 24 h	Old 24 h		Young 72 h		Old 72 h	
TNFα	165 / 20	340 / 25	**a**	63 / 28	**b**	230 / 50	**a,b**
IL-6	437 / 56	1591 / 282	**a**	1044 / 381		6190 / 1428	**a,b**
MCP-1	20 / 3	201 / 53	**a**	53 / 14		269 / 43	**a**
CXCL1	686 / 116	1038 / 112		709 / 212		1300 / 242	**a**
MIP-1α	82 / 8	353 / 32	**a**	53 / 14		120 / 23	**a,b**
IL-12	18 / 5	0 / 0	**a**	25 / 13		84 / 27	**a,b**
IFNγ	719 / 205	4 / 1	**a**	346 / 95		326 / 80	**b**
IL-10	7 / 1	10 / 2		9 / 2		14 / 3	
IL-17A	7 / 2	2 / 0		14 / 9		78 / 32	**a,b**

**B)Serum Cytokines**	**Young 24 h**	**Old 24 h**		**Young 72 h**		**Old 72 h**	

TNFα	10.0 / 0.8	12.1 / 3.0		7.2 / 1.8		8.1 / 2.0	
IL-6	45.7 / 7.2	40.9 / 7.9		21.0 / 8.4		54.9 / 17.1	
IL-12	1.8 / 0.5	0.2 / 0.1	**a**	1.7 / 0.5		1.5 / 0.7	
IFNγ	16.8 / 5.6	0.1 / 0.0	**a**	5.3 / 1.9	**b**	1.5 / 0.4	

Cytokine expression in the BALF (A) and in the serum (B) in young and old mice after 24 h and 72 h of LPS exposure (pg/mL). Note: Cytokine expression in control (saline) mice was below the detection limit of the assays. Values are displayed as mean / SEM; a = significant (p<0.05) age effect within same exposure time and b = significant exposure time effect within same age group.

## RESULTS

### Pro-inflammatory signaling in ALI is aggravated in the lungs of old compared with young mice

**Figure 3:**
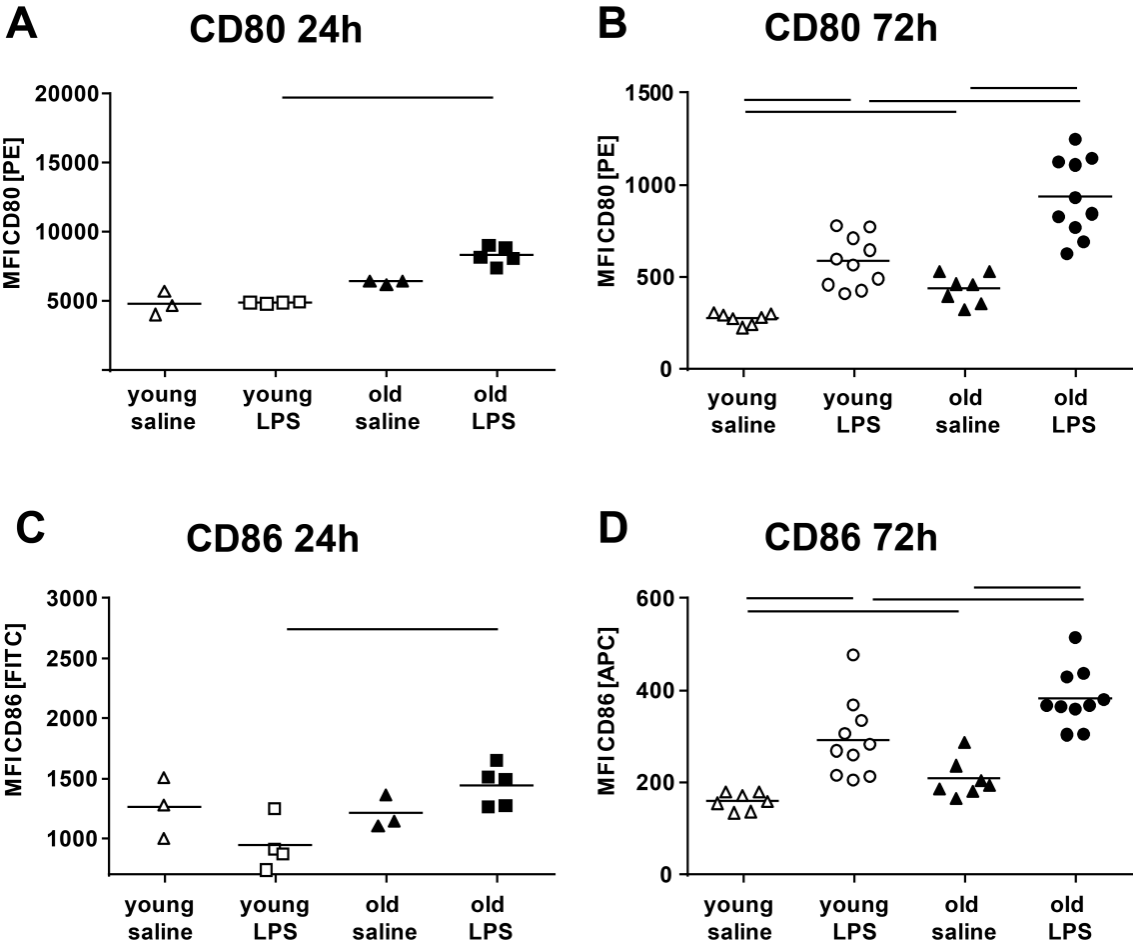
Activation of BALF AM after 24 h and 72 h exposure time CD80 expression (**A** and **B**) and CD86 expression (**C** and **D**) were assessed in BALF AM from young and old control and LPS mice after 24 h and 72 h of exposure time. AM of old mice showed enhanced CD80 and CD86 expression compared with young mice after LPS exposure at both time points. Note: 24 h and 72 h experiments are not directly comparable since two protocols with different staining compensations were applied. Data points from 24 h experiments each represent the result of two pooled BALF samples (see material and methods). Means are expressed by horizontal bars; lines indicate statistically significant (*p* < 0.05) differences between groups.

Intranasal administration of LPS caused an infiltration of inflammatory cells in the alveolar and peri-bronchiolar/-vascular area as displayed by histopathology (Fig. 1). The inflammatory cells were mainly neutrophilic granulocytes with lesser numbers of macrophages and were more abundant in old compared with young mice. The cellular infiltration did not change between 24 h and 72 h post-exposure in the young mice; however, an obvious increase became apparent in the old mice over time. No septal fibrosis was apparent in young or old mice, but pulmonary edema [9], which is common for LPS models of ALI [18]. In line with pulmonary histopathology, old LPS-mice showed significantly more BALF protein (Fig. 2A), total BALF cells (Fig. 2B) and BALF neutrophils (Fig. 2C) compared with young LPS-mice and the number of cells further increased by 50% from 24 to 72 h post- exposure in old mice. At 72 h post-exposure, the old mice also had significantly more BALF macrophages compared with young mice (Fig. 2D) and both, young and old mice showed an increase in BALF lymphocytes (Fig. 2E). The higher BALF protein concentration in old compared with young LPS-mice after both exposure time points (Fig. 2A), suggests an enhanced edema formation in the old mice as shown previously [9]. Activation of alveolar macrophages (AM) in the BALF was assessed by flow cytometric measurement of M1 markers CD80 and CD86 (Fig. 3). A significant increase in CD80 and CD86 expression was measurable in AMs of young and old mice after 72 h exposure time. The activation was significantly stronger in AM from old LPS-mice and already apparent after 24 h.

Measurements of BALF cytokines indicate an enhanced pro-inflammatory response in old compared with young mice in ALI (Table 1A). Cytokine levels of control animals were below the detection limit of the assay. Pro-inflammatory cyto- and chemokines such as TNFα, IL-6, MCP-1 and MIP-1α were significantly elevated in the old animals 24 h and 72 h after induction of ALI and expression levels of CXCL1 after 72 h. Over time, the expression of TNFα declined in both age groups, whereas the expression levels of IL-6 showed a significant, 4-fold increase in the old mice. IFNγ and IL-12 were only expressed in the BALF of young mice after 24 h of LPS exposure, but increased in old mice to a similar (IFNγ) or higher (IL-12) level as in young mice within 72 h. Only a low expression level with no difference between all LPS-groups was detected for IL-10 and a significant increase in IL-17 of old mice 72 h after LPS exposure.

### Systemic inflammatory response in ALI is not enhanced in old compared with young mice

**Figure 4:**
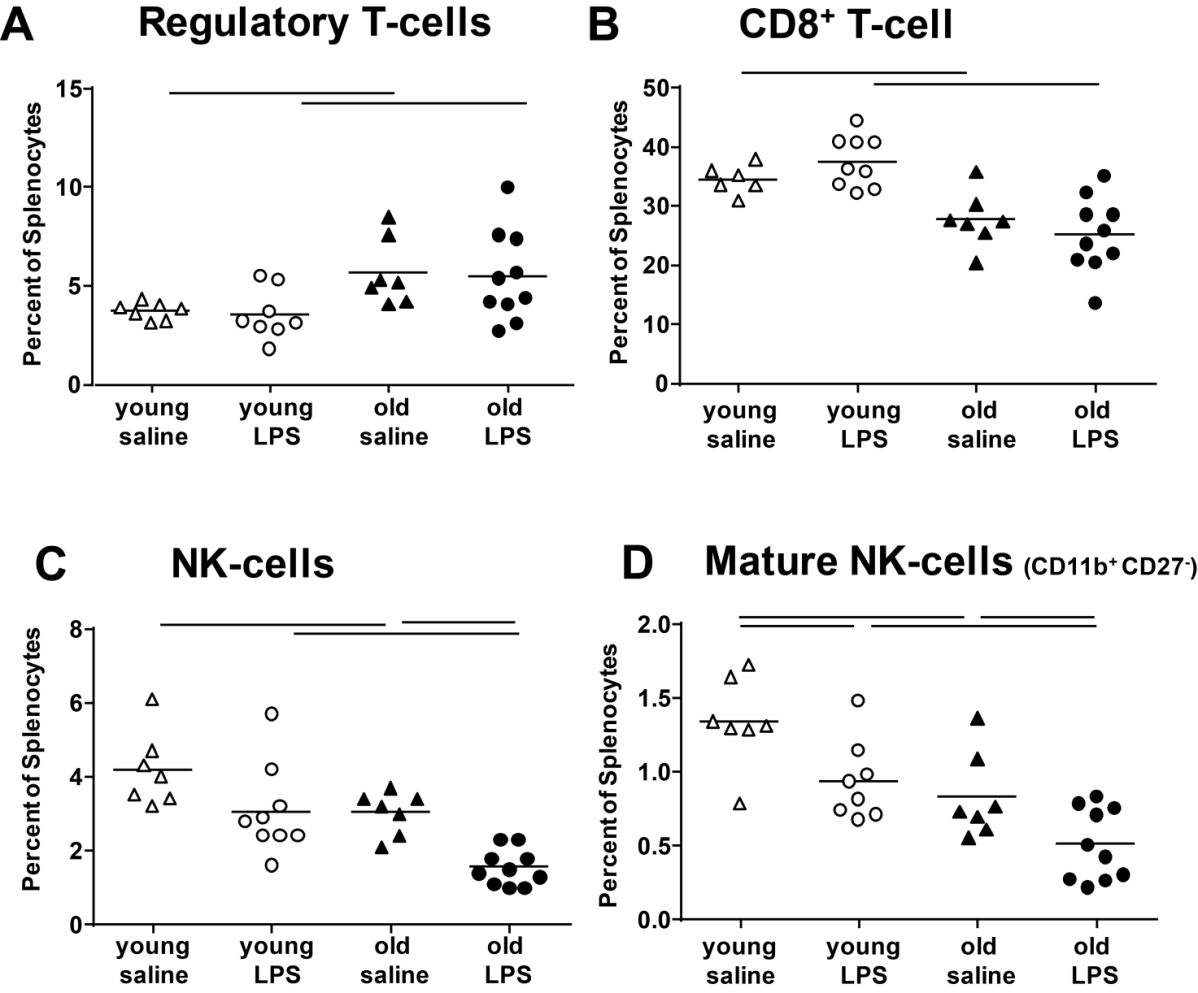
Lymphocyte populations in the spleen Lymphocyte populations were assessed in spleens of control and LPS-mice 72 h after LPS exposure. Percentage of CD25^+^ T-regs was elevated in the spleens of old mice (A), whereas CD8^+^ T-cells and NK cells were reduced (**B** and **C**). No significant changes were detected with LPS exposure in the percentages of T-regs and CD8^+^ T-cells. However, a decline with LPS exposure was measured in percentage of total NK cells in old mice and mature NK cells in both age groups (**D**). Each data point represents one animal; means are expressed by horizontal bars; lines indicate statistically significant (*p* < 0.05) differences between groups.

**Figure 5:**
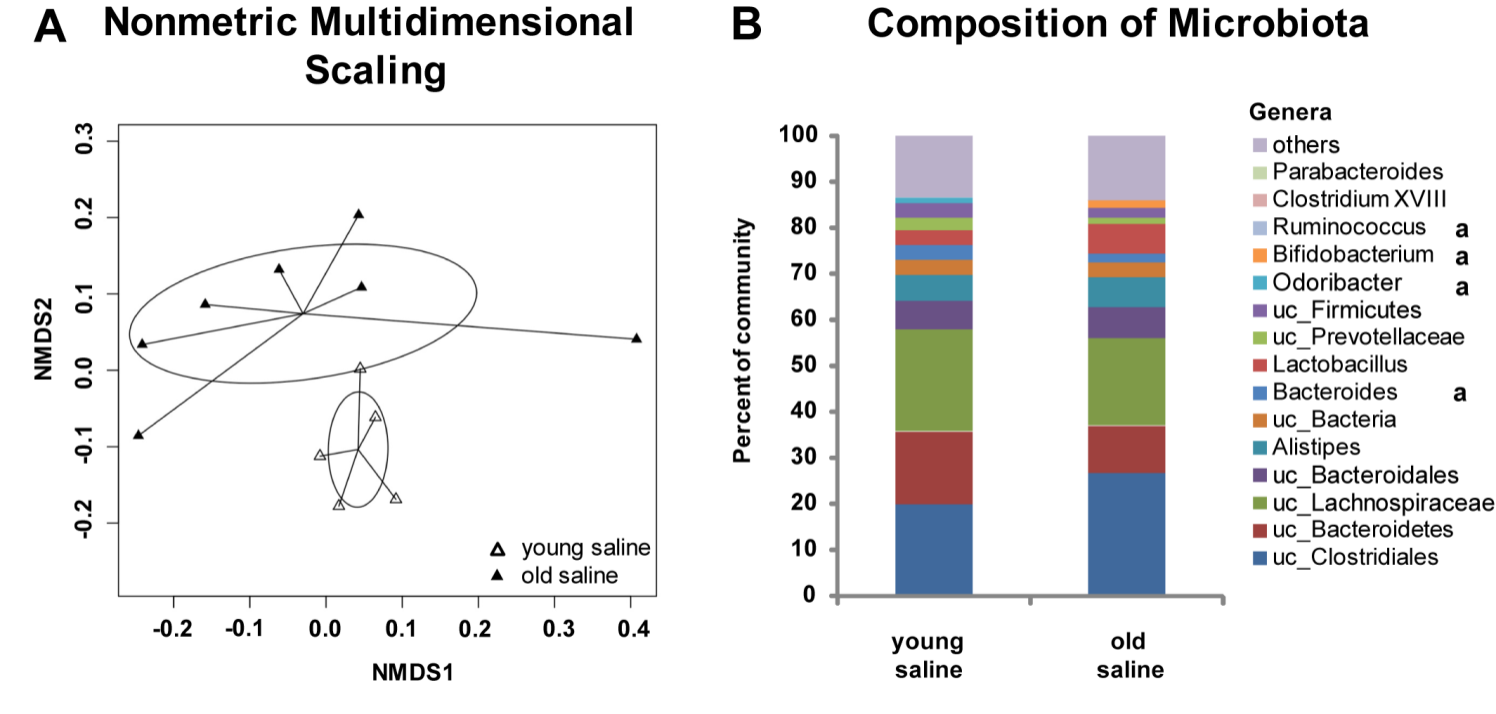
Composition of gut microbiota Caecal microbiota composition was analyzed in young and old control mice. Nonmetric multidimensional scaling (NMDS) of bacterial communities for young mice (empty triangle) and old mice (filled triangle) are shown (**A**). Age significantly influenced community structure based on permutational ANOVA analysis (*p*<0.01). Ellipses represent the 95% confidence interval on standard errors of points. The composition of the caecal bacterial community at genus level (**B**) is given with specific taxa that significantly differed with age (**a**) based on FDR corrected bootstrapped Mann-Whitney U tests). uc: unclassified.

Changes in systemic inflammation were assessed by blood serum cytokine, spleen cell population (CD8^+^ T-cells, T-regs and NK cells) analysis and examination of the gut microbiome. Data of cytokine levels in the blood are shown in table 1B. Although there was a significant increase in pro-inflammatory cytokines in the serum of mice with ALI (TNFα and IL-6), no significant age and exposure time-dependent differences were measured. IL-10 expression was below the detection level in all exposure groups. The main age effects detected in the serum were the lower levels of IL-12 and IFNγ in the old mice after 24 h of exposure time. This observation led to the analysis of lymphocytes, as potential producers of systemic or local IFNγ and stimulators of adaptive immunity; analyzed in the spleen as a secondary lymphatic organ. Levels of T-regs where elevated in old mice (Fig. 4A), whereas levels of CD8^+^ T-cells and NK cells were reduced in the spleen of old mice (Fig. 4B and 4C). No significant changes were detected with LPS exposure for T-regs and CD8^+^ T-cells, but a decline in total NK cells of old mice and in mature NK cells (CD11b^+^ and CD27^-^, Fig. 4D) of both young and old mice after LPS exposure. The community structure of the gut microbiome was significantly distinct between young and old mice with many taxa significantly differing with age (Fig. 5). The overall *Firmicutes* to *Bacteroidetes* ratio was 1.8±0.6 and 2.4±0.8 in young and old mice, respectively.

### Alveolar but not bone marrow derived macrophages show enhanced inflammatory activity with progressing age

**Figure 6:**
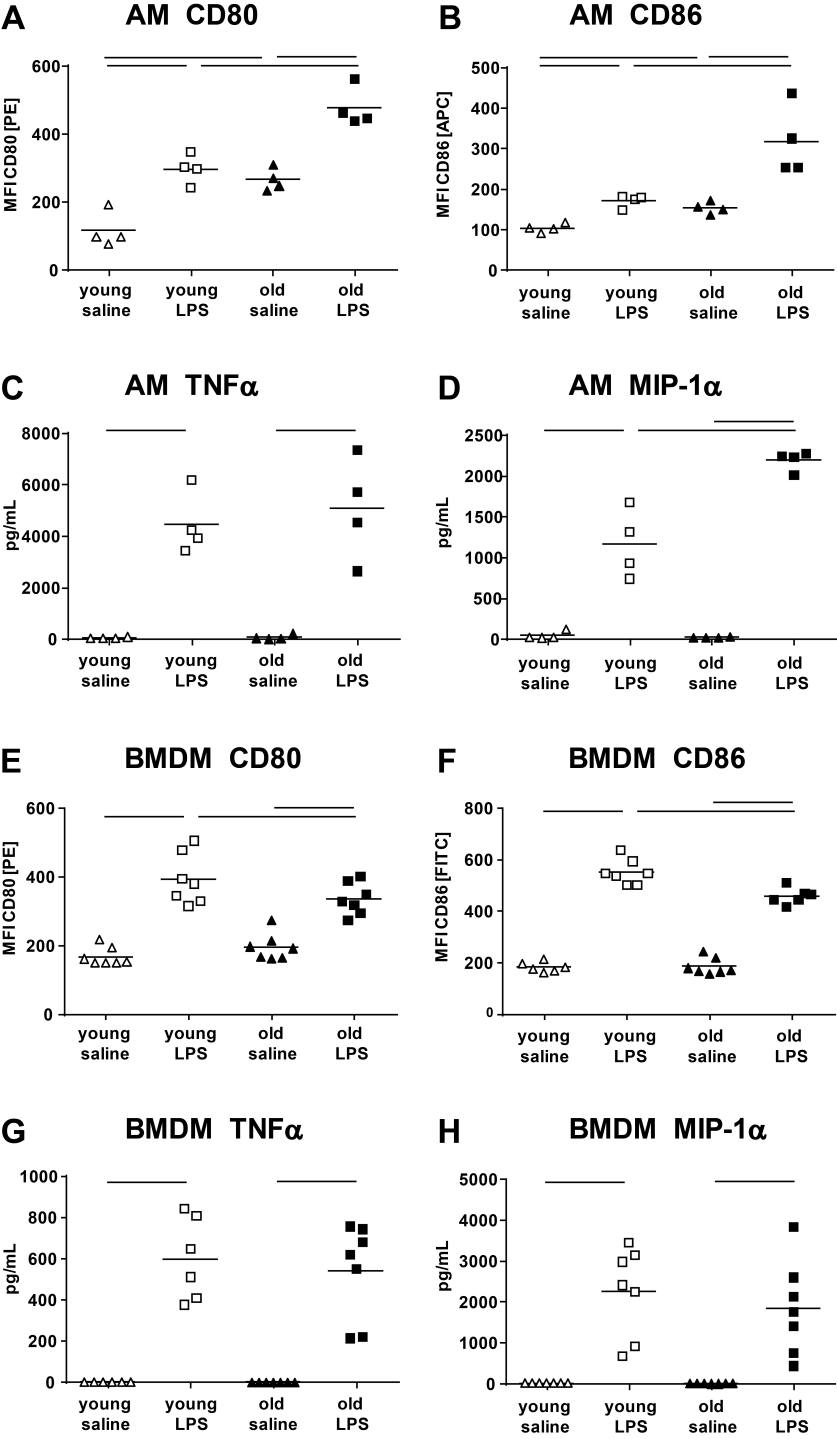
*In vitro* stimulation of AM and BMDM AM and BMDM were stimulated for 24 h with 100 ng/mL LPS. CD80 and CD86 expression of *in vitro* stimulated AM was greater in cells from old compared with young donors (**A** and **B**). No difference was measured in TNFα secretion in the supernatant, but enhanced levels of MIP-1α in AM from old mice (**C** and **D**). In comparison, CD80 and CD86 expression was lower in LPS exposed BMDM from old compared with young mice (**E** and **F**). No difference was measured in cytokine expression in old compared with young BMDM (**G** and **H**). Each data point indicates an individual measurement; means are expressed by horizontal bars; lines indicate statistically significant (*p*<0.05) differences between groups.

Since AM of old mice were more activated 24 and 72 h after LPS exposure, macrophage specific contributions were further investigated *in vitro*. *In vitro* stimulation of AM with LPS resulted in an increased expression of CD80 and CD86 which was significantly greater in AM isolated from old compared with young mice (Fig. 6A and B). MIP-1α expression was significantly elevated in the cell culture supernatant of old compared with young AM after LPS exposure (Fig. 6D). However, no age-related differences were detected in the expression of TNFα (Fig. 6C) or other cytokines such as CXCL1, IL-6 or MCP-1 (data not shown). BMDM also showed elevated expression of CD80 and CD86 (Fig. 6E and 6F) after stimulation with LPS, but no enhanced activation in cells from old mice. In contrary, BMDM from old mice showed less CD80 and CD86 expression compared with those from young mice. No age-dependent differences were detected for TNFα and MIP-1α expression in the supernatant of LPS-exposed BDMD (Fig. 6G and 6H), also not for CXCL1, IL-6 or MCP-1 (data not shown).

## DISCUSSION

In the current study we investigated the cellular, pulmonary and systemic inflammatory response in ALI of young and old mice. An enhanced pro-inflammatory signaling und neutrophil recruitment was predominant in the lungs of old mice. As a consequence the old mice suffered from greater pulmonary damage with more edema and inflammatory cell infiltrates, which causes age-related changes in pulmonary structure and function, as shown in our previous study [9]. Whereas the pro-inflammatory signaling started declining in young mice between 24 h and 72 h after LPS application, it further progressed in old animals. In addition, two of the old LPS-mice died within 72 h post-exposure, indicating that the greater pulmonary inflammation is related to a worse prognosis and enhanced mortality in old mice with ALI.

### Pulmonary inflammatory response and activation of alveolar macrophages

The greater acute inflammatory response in the old mice of this study was characterized by enhanced expression levels of TNFα, IL-6, MCP-1, CXCL1 and MIP-1α in the BALF. These cytokines are mediators of the acute inflammatory response and involved in the activation and recruitment of leukocytes to the site of inflammation, which is in line with the increased number of neutrophils and monocytes in BALF and lung tissue of old mice. However, not all cytokines were elevated in old compared with young LPS-mice. For example IL-10, an anti-inflammatory cytokine, was equally induced in young and old LPS-mice and IFNγ and IL-12, related to the activation of the adaptive immunity and microbial elimination, were even significantly lower in the BALF of old compared with young mice 24 h after LPS exposure. Thus old mice did not show a global increased reaction towards LPS, but an elevated pro-inflammatory signaling in the lung.

AMs are some of the first immune cells of the lung that respond to pathogen or danger associated molecular pattern (DAMP/PAMP) signaling and initiate inflammatory reactions. They secrete cytokines and chemokines for leukocyte recruitment and participate in phagocytosis and antigen presentation. In our study we measured the activation of AM by assessing the expression of CD80 and CD86. These markers are considered as M1 markers of the classically activated macrophages that are usually observed after LPS stimulation and in early inflammatory response [27,28]. Furthermore they have been suggested as biomarkers for severity in sepsis [29]. Along with the elevated BALF cytokine profile we found an increased activation of AM in old mice after LPS stimulation. To clarify if AM of old mice show a greater activation potential per se or if the pulmonary environment stimulates an increased reactivity, we isolated AM and BMDM from young and old mice and stimulated them with LPS* in vitro*. Results of the *in vitro* stimulation revealed that AM of old mice became more activated compared with the ones from young mice - as observed *in vivo*. Characterization of the cytokine profile of AM showed that the old macrophages release significantly more MIP-1α. MIP-1α is a cytokine that is involved in leukocyte recruitment to the site of inflammation [30], thereby contributing to migration of inflammatory cells to the lungs in ALI. The finding that isolated AM show a similar activation as *in vivo,* hence supports the hypothesis that AM play a key role in pro-inflammatory signaling and age-related aggravation of ALI.

In contrast to AM, *in vitro* stimulation of BMDM did result in lower activation and expression of CD80/CD86 in cells isolated from old mice and cytokine profiles did not differ with age upon LPS stimulation. BMDM show features of monocyte-like-macrophages, as recruited during inflammation. AM, however, are highly specialized tissue resident macrophages that form a unique, self-renewing population in the lung [31,32]. These two cell types therefore represent different populations of macrophages. Previous studies on age-related changes in macrophage function give evidence that effects of aging may differ within macrophage populations. For example a study with macrophages isolated from the spleen indicated a greater activation of young compared with old macrophages after LPS exposure *in vitro* [33], similar to the results detected in BMDM. However, AM from old compared with young mice that were exposed to *Mycobacterium tuberculosis* showed a greater response [34], like the AM in our study* in vivo* or *in vitro*. Senescence-related changes in cell phenotypes have been described as a hallmark of aging [35] and depending on the cell type and its niche, the impact of aging varies [36]. In this context it has also been suggested that characteristics of senescence differ within macrophage populations with effects on inflammatory signaling, bacterial clearance or antigen-presentation [37,38]. Different mechanisms such as exhaustion of progenitor cells or cell stress could lead to senescence-related alteration in macrophages and future studies will be performed to investigate the mechanisms of AM aging and their contributions in pulmonary disease.

### Systemic inflammatory response with age and LPS

Comparisons in systemic responses with age and LPS were further assessed by analyzing cytokine expression in the blood serum, splenocyte populations and the gut microbiome. No age-related difference was measured for TNFα and IL-6 expression in the serum, suggesting that the enhanced pro-inflammatory response, as detected in the lungs of old mice, was not present systemically. The expression pattern of IFNγ and IL-12 was similar in the serum and in the BALF. Twenty-four hours after LPS exposure, only the young mice showed a solid expression of IFNγ and IL-12 in the serum that evened out 72 h post-exposure. IL-12 is produced by a variety of leukocytes. It stimulates the cytotoxic activity of NK cells and CD8^+^ T-cells and the secretion of IFNγ [39]. IFNγ, otherwise, is secreted by T-cells and NK cells and stimulates macrophages to release IL-12, express MHCII and participate in antigen presentation as well as phagocytosis of bacteria [39,40]. In line with the lower levels of IFNγ in the old LPS animals, an age-dependent decline in total and mature NK cells and CD8^+^ T-cells was measured in the spleens of old mice. It is therefore possible that a delayed expression of IFNγ and IL-12 is related to a decline in NK cells and CD8^+^ T-cells with age. Whereas the percentage of CD8^+^ T-cells did not change with LPS exposure, a significant decrease in mature NK cells was found in LPS exposed mice. The decrease was relatively similar in both age groups and could be the result of cell recruitment to the lungs. We did observe an increase in BALF lymphocytes at 72 h post-exposure, but we did not quantitate NK and T-cell populations in the lung, as their populations are generally very low and require a large amount of tissue for proper analysis. However, a decline in CD8^+^ T-cells and total or mature NK cells with advanced age has been described previously [22,41,42]. The lower percentage of CD8^+^ T-cells and NK cells with age could therefore indicate a general feature of immunosenescence with impact on activation of adaptive immunity and resolution of infectious disease. This could be particularly relevant for viral infections like influenza mediated pneumonia.

In the spleen we further found an age-dependent increase in T-regs, but no changes in splenic T-reg populations were detected with LPS exposure. T-regs are a main source of IL-10. In a previous study from Williams and colleagues a shortage of IL-10 in old mice was identified as the central cause of age-related severity in *Streptococcus pneumoniae* derived ALI [13]. However, in our study no age-related differences were detected in BALF IL-10 expression and expression levels in the blood serum were below the assay detection limit - despite the greater number of splenic T-regs in old mice. Based on these findings we do not consider a lack of anti-inflammatory IL-10 as a main cause for the pro-inflammatory response in the old animals of our study.

The gut microbiome has also been suggested to influence the pulmonary immunity including development of pneumonia and other pulmonary disease [19,20] - for example by modulating T-reg populations in the intestine [43] and thereby affecting the host immunity including the lung [44]. Age-related changes of the gut microbiome and its contribution to inflamm-aging have been reported previously [45,46]. Also in this study we found differences in the gut microbiome between young and old mice along with age-related differences in splenic lymphocyte populations, supporting the idea that the composition of gut bacteria affects systemic immunosenescence. However, our understanding of the influence of the gut microbiota in aging is still in its infancy and more function-based research is needed to fully reveal its role in immunosenescence and pulmonary disease. Age-related changes in the lung microbiome would further be of interest, but have, to the best of our knowledge, not been investigated so far. Analysis of the lung microbiome is prone to sample contamination and particularly challenging in the mouse lung where sample volumes are very small. Therefore we did not asses microbial changes in the lung with age and ALI in this current investigation.

### Summary and conclusion

The characterization of systemic and local inflammatory responses in ALI with progressing age is relevant to improve age-related therapeutic approaches. Our study shows that immunosenescence in ALI is associated with an enhanced pro-inflammatory signaling that originates from the lung. AM, the first responders to PAMP recognition and inflammatory signaling, were more activated in the old mice - *in vivo* and *ex vivo*, suggesting their active contribution to immunosenescence and the pathology of ALI in aging. A decline in NK cells and CD^8+^ T-cells in the spleen with age as well as a delayed response in IFNγ and IL-12 further indicate a decrease in activation of adaptive immunity with advancing age that could further contribute to a worse prognosis of the elderly with ALI.
